# Revisiting the Warburg Effect with Focus on Lactate

**DOI:** 10.3390/cancers14246028

**Published:** 2022-12-07

**Authors:** Eva Kocianova, Viktoria Piatrikova, Tereza Golias

**Affiliations:** 1 Department of Tumor Biology, Institute of Virology, Biomedical Research Center, Slovak Academy of Sciences, 84505 Bratislava, Slovakia; 2 Department of Molecular Biology, Faculty of Natural Sciences, Comenius University, 84215 Bratislava, Slovakia

**Keywords:** Warburg effect, glucose metabolism, lactate, lactate dehydrogenase, lactate shuttle, lactate oxidation, lactate signaling, lactylation

## Abstract

**Simple Summary:**

Almost a century ago, Nobel Prize laureate Otto Warburg realized that cancer cells consumed much more glucose than normal cells and also produced large amounts of lactate even in aerobic conditions, which was very surprising at the time. After decades of research, it eventually became clear that lactate was much more than just a waste product and that cancer cells were programmed to use it to their advantage. In this review, we discuss the current state of knowledge regarding the purpose of lactate in cancer and how our understanding of its significance has evolved over time.

**Abstract:**

Rewired metabolism is acknowledged as one of the drivers of tumor growth. As a result, aerobic glycolysis, or the Warburg effect, is a feature of many cancers. Increased glucose uptake and glycolysis provide intermediates for anabolic reactions necessary for cancer cell proliferation while contributing sufficient energy. However, the accompanying increased lactate production, seemingly wasting glucose carbon, was originally explained only by the need to regenerate NAD^+^ for successive rounds of glycolysis by the lactate dehydrogenase (LDH) reaction in the cytosol. After the discovery of a mitochondrial LDH isoform, lactate oxidation entered the picture, and lactate was recognized as an important oxidative fuel. It has also been revealed that lactate serves a variety of signaling functions and helps cells adapt to the new environment. Here, we discuss recent findings on lactate metabolism and signaling in cancer while attempting to explain why the Warburg effect is adopted by cancer cells.

## 1. Introduction

The hallmarks of cancer concept coined by Hanahan and Weinberg two decades ago has evolved over the years, currently incorporating up to 14 underlying cellular parameters that accompany carcinogenesis [1]. Reprogrammed metabolism has a firm position among them and is considered a core hallmark of cancer. Cancer cells rewire their metabolism in response to the tumor microenvironment and their proliferative needs. They alter the flux of metabolites through various metabolic pathways, so the modulated metabolism provides energy and substrates essential for growth and cell proliferation. Interestingly, there are many similarities between the cancer metabolic phenotype and that of hypoxia, ischemia, embryonic growth and development, exercise, obesity, diabetes, viral infection, immune response, and more. Due to this fact, a plethora of studies exist that uncover new fundamental metabolic pathways and even completely reshape our understanding of historically established metabolic processes. One such example is the previously unfair and incorrect view of lactate—originally seen as a waste product of anaerobic metabolism and now appreciated as a major fuel formed even under fully oxygenated conditions [2]. In this review, we focus on the enzyme lactate dehydrogenase (LDH) that catalyzes the reversible conversion of pyruvate to lactate and thus serves as an important link between oxidative metabolism and glycolysis. We also reflect on its previously believed strictly cytosolic activity, as mitochondrial LDH localization has been established [3,4,5]. Additionally, we evaluate in more detail the main objective of LDH activity apart from pyruvate/lactate production, i.e., the recycling of NADH/NAD^+^, and its significance for cancer cell metabolism.

## 2. Glucose Metabolism in Cancer—Warburg Effect

**Figure 1:**
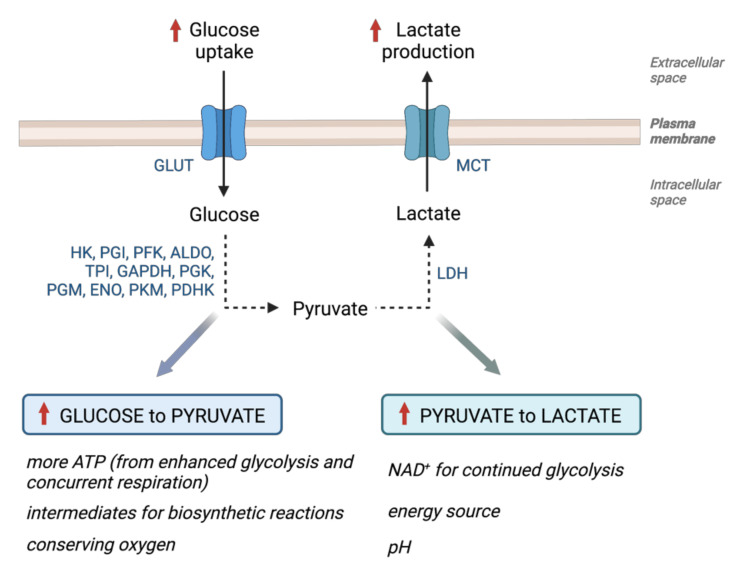
Advantages of the Warburg effect for cancer cells. Cancer cells benefit from increased glucose uptake, glycolysis, and lactate production in several ways (listed in *Italic*). The HIF transcription factor upregulates the expression of many isoforms of genes involved in the Warburg effect (in blue) (GLUT—glucose transporter; HK—hexokinase; PGI—phosphoglucose isomerase; PFK—phosphofructokinase; ALDO—aldolase; TPI—triosephosphate isomerase; GAPDH—glyceraldehyde-3-phosphate dehydrogenase; PGK—phosphoglycerate kinase; PGM—phosphoglycerate mutase; ENO—enolase; PKM—pyruvate kinase; PDHK—pyruvate dehydrogenase kinase; LDH—lactate dehydrogenase; MCT—monocarboxylate transporter).

Rapidly proliferating cancer cells have quantitatively, and qualitatively different metabolic requirements compared to normal non-dividing cells. Even cancer cells within a tumor may have different metabolic needs due to their local microenvironments and available nutrient supplies. The most common changes in tumor metabolism are associated with the metabolism of glucose and glutamine, biosynthesis of lipids, and the tricarboxylic-acid (TCA) cycle. The delicate balance between these pathways fine-tunes the adaptive response of cancer cells. The biggest number of studies on cancer metabolism is by far concentrated on glucose because of Otto Warburg’s groundbreaking discovery in the 1920s [6]. He observed that cancer cells had higher glucose uptake in comparison to non-transformed cells, and instead of using it primarily to produce ATP by mitochondrial respiration and oxidative phosphorylation (OXPHOS), they mainly metabolized glucose via pyruvate to lactate by LDH regardless of the presence of oxygen (coined as the Warburg effect by Efraim Racker in 1972 [7]). The reason why cancer cells behave in this manner has been the subject of much debate (Figure 1).

Warburg originally proposed that aerobic glycolysis was a consequence of defective mitochondria making oxidative metabolism non-functional, a statement he later corrected [8]. The cancer metabolism scientific community largely accepted that cancer cells chose a less-efficient method of extracting energy from glucose (2 ATP molecules from glycolysis vs. 36 ATP from OXPHOS) in exchange for gaining necessary intermediates for anabolic reactions important in cell growth [9]. However, the work of Otto Warburg actually showed that even energy-wise, cancer cells produced 10–13% more ATP than normal cells thanks to the approximately 10 times higher glucose uptake that allowed them to simultaneously carry out glycolysis and mitochondrial respiration, while even in the absence of oxygen, the enormous glycolytic flow yielded 2/3 of the ATP that normal cells produced by respiration [10,11]. In general, cancer cells gain 40–75% of their energy from glycolysis, and the remainder is synthesized in respiring mitochondria through OXPHOS [12]. Their mitochondria are thus vital and contribute to ATP production and the supply of biosynthetic intermediates [13,14].

The precise molecular mechanism that triggers the Warburg effect in cancer remains unclear, although tumor suppressors (p53) and oncogenes (SRC, AKT, RAS) all seem to converge on the hypoxia-inducible transcription factor HIF or the oncogenic transcription factor MYC. Accordingly, all the glycolytic enzymes have isoforms that are HIF target genes with hypoxia-response elements (HRE) in their promoters [15] (Figure 1). HIF is degraded in normoxic conditions and conversely stabilized in hypoxia [16,17]. Tumor hypoxia is a common phenomenon and a bad prognosis factor for cancer patients; however, HIF can be stabilized even under normoxia and initiate the hypoxic response in fully aerobic conditions. In normoxia, prolyl hydroxylases (PHDs) use molecular oxygen to hydroxylate the alpha subunit of HIF, targeting it for recognition by the von Hippel-Lindau tumor suppressor (VHL) and subsequent degradation in the proteasome. Mechanisms, such as mutations in the VHL gene in some cancers (clear cell renal cell carcinoma) or accumulated alpha-ketoglutarate or succinate or even lactate that inhibit PHDs, make it possible for HIF to escape normoxic degradation and induce expression of its target genes regardless of oxygen tension [18,19,20,21,22]. Glucose transporters (GLUT) 1 and 3 were one of the first genes identified as HIF targets, coinciding with their role in increasing glucose uptake as a prerequisite for the Warburg effect [23,24,25]. Augmented lactate production in tumors, as observed by Warburg, is the final reaction of glycolysis, also catalyzed by a HIF-target gene, lactate dehydrogenase A [26,27]. It is equally important for the Warburg effect because while converting pyruvate to lactate, it facilitates the continuous glycolytic flow by regenerating NAD^+^ from NADH produced in the preceding glycolytic reactions.

**Figure 2:**
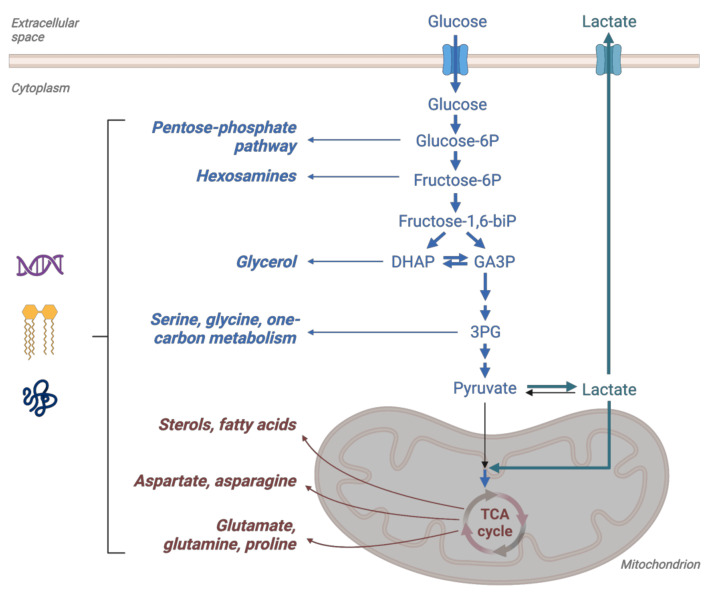
Glucose metabolism as a source of biosynthetic intermediates. The breakdown of glucose through glycolysis (blue) and the TCA cycle (brown) provides the building blocks necessary for the synthesis of nucleotides, lipids, and proteins (adapted from [34]).

As mentioned above, the boosted glycolytic flow was thought to fuel the biosynthetic needs of proliferating cancer cells through essential metabolic intermediates, such as glucose-6-phosphate, glyceraldehyde-3-phosphate, 3-phosphoglycerate, etc. (Figure 2). However, only 10% of the glucose carbon is used in biosynthetic reactions, and 90% is consumed in the production of lactate or alanine [28]. The TCA cycle localized in the mitochondrial matrix also plays a role in providing building blocks for the synthesis of macromolecules. Two main substrates for oxidative metabolism are pyruvate (generated from glucose or lactate) and glutamine. Pyruvate is converted to acetyl-coenzyme A (acetyl-CoA) by the pyruvate dehydrogenase complex (PDH) in the mitochondrial matrix. This step is regulated by pyruvate dehydrogenase kinases (PDHK) that inhibit the PDH complex by phosphorylation. Incidentally, PDHK1 and PDHK3 are also HIF-target genes that support glycolysis by decreasing mitochondrial function [29,30]. The fate of glucose is, therefore, mainly decided by the action of PDH, PDHK, and LDH. Another advantage of aerobic glycolysis and the action of PDHKs has reduced oxygen consumption, which could conserve oxygen for less oxygenated cancer cells, benefiting the tumor as a whole [31]. Equally, the production of lactate by glycolytic cells benefits more oxygenated tumor cells that use it to fuel their TCA cycle [32]. It is not surprising that monocarboxylate transporter 4 (MCT4) that exports lactate also belongs to the group of hypoxia-regulated HIF target genes [33]. Along with the lactate anions, protons are co-transported through MCT4, which helps with the maintenance of neutral intracellular pH but leads to extracellular acidosis, toxic for the surrounding non-transformed cells, while also promoting an invasive phenotype and the formation of metastases—a further consequence of the Warburg effect.

## 3. Lactate Dehydrogenase

LDH is a highly active and final glycolytic enzyme that belongs to the 2-hydroxy acid oxidoreductase family. As already mentioned, it plays a key role in the switch of oxidative metabolism to glycolysis and also creates a balance between carbohydrate catabolism and anabolism. During the conversion of pyruvate to lactate, the regeneration of NADH to NAD^+^ occurs, which is essential for ongoing glycolysis. In addition, LDH is involved in the process of gluconeogenesis, where lactate is metabolized to glucose through a reverse LDH reaction. Furthermore, lactate oxidation can also feed the TCA cycle (more in Section 4). High levels of LDH in the blood, on the other hand, may indicate pathological conditions in the body, such as traumatic injury, liver disease, certain types of anemia, heart attack, viral infection, and cancer [35].

### 3.1. LDH Isozymes

**Figure 3:**
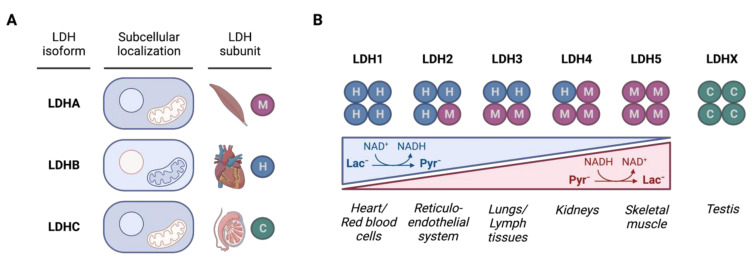
LDH isoforms and tetramers. (**A**) LDHA isoform, also known as the muscle (M) subunit, is localized predominantly in the cytoplasm and partly in the nucleus; LDHB isoform, also known as the heart (H) subunit, is localized in mitochondria and the cytosol; the subcellular localization of the testis-specific LDHC isoform is similar to that of the LDHA isoform (source GeneCards). (**B**) Tetramerization of the LDH subunits forms LDH isozymes with different affinities to substrate and organ distribution (adapted from [39,44,45,46]).

LDH is prevalent in a variety of organisms, such as plants, animals, and humans. There are only a few changes in the amino acid sequences among species [36]. LDH is a homotetrameric or heterotetrameric molecule consisting of two subunits—M (muscle) and H (heart)—that are distributed in the organism based on the specific metabolic needs of tissues (Figure 3). The M subunit is abundant in skeletal muscles, where it switches oxidative metabolism to glycolysis during physical activity. On the other hand, the H subunit is dependent on aerobic metabolic pathways and is mainly present in the heart [37]. The myocardium needs a continuous supply of energy, which is maintained by the conversion of lactate to pyruvate. The maximal activity of the H subunit is in low pyruvate concentrations and is inhibited by the abundance of pyruvate. Conversely, the M subunit is active in the presence of high concentrations of pyruvate [38]. In humans, the M subunit, known as LDHA, is encoded by the *ldha* gene located on chromosome 11, while the H subunit, known as LDHB, is encoded by the *ldhb* gene located on chromosome 12. Combinations of the M and H subunits form 5 isozymes LDH1-LDH5 (Figure 3B). LDH isozymes have different affinities to the substrate, inhibition concentrations, isoelectric point, electrophoretic mobility, expression, and tissue specificity. LDH1 is a homotetramer composed of four identical H subunits and is preferentially localized in the heart and red blood cells. The heterotetramer LDH2 consists of three H subunits and one M subunit and is mainly present in the reticuloendothelial system. Isozyme LDH3 has two M and two H subunits and is present in the lungs and lymph tissue. LDH4 consists of one H and three M subunits and is the major isoenzyme of the kidneys. LDH5 is a homotetramer consisting of four identical M subunits and is present in skeletal muscles [39]. Tetramer formation is regulated both by the abundance of substrate, as well as by post-translational modifications [40]. Apart from the *ldha* and *ldhb* genes, there is also the *ldhc* gene that encodes subunit LDHC on chromosome 11 that creates a homotetrameric molecule of the testis-specific lactate dehydrogenase C, also known as LDHX [41,42]. In vertebrates, the occurrence of the *ldhd* gene has also been reported. It encodes lactate dehydrogenase D (LDHD) that converts the D-isomer of lactate and is associated with pathways not connected to glucose metabolism [43].

### 3.2. LDH Structure

**Figure 4:**
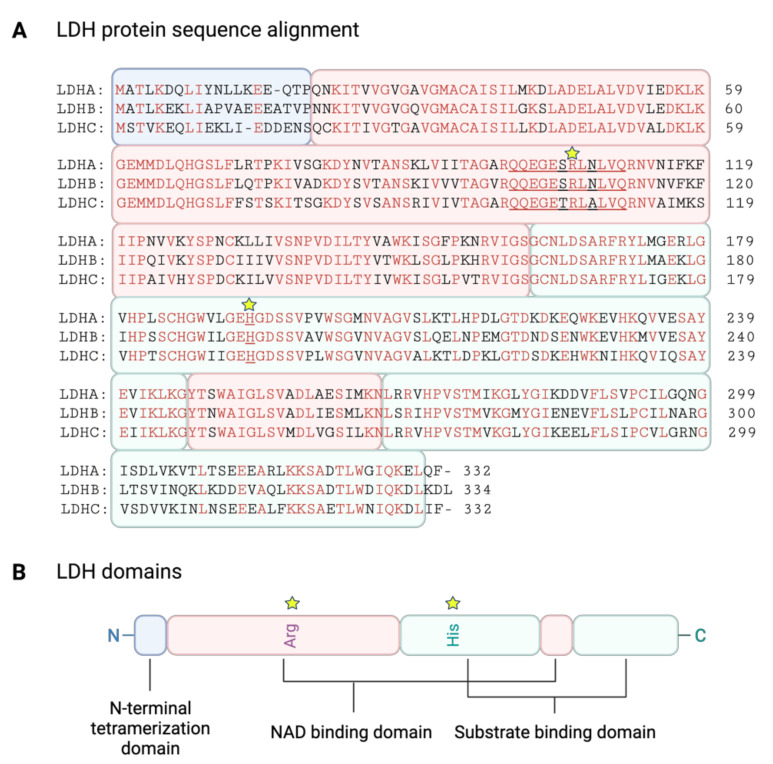
LDH protein sequence alignment and domains. (**A**) Clustal Omega alignment of human LDHA, LDHB, and LDHC amino acid sequences. (Identical amino acids shared among all three isoforms are depicted in red. Stars highlight essential arginine—R106 and catalytic histidine—H193. Amino acids forming the mobile loop are underlined. Different background colors designate the same LDH protein domains as depicted in (**B**)). (**B**) LDH protein domains with highlighted Arg106 and His193 are essential for LDH activity (adapted from [40]).

In humans, the sequence similarity between LDHA and LDHB is 75%, between LDHA and LDHC 74%, and 69% between LDHB and LDHC [47] (Figure 4). More than 90% of the protein is made up of the nucleotide- and substrate-binding domains. The remaining amino acids at the N-terminus interact with the C-terminus of a neighboring monomer and are critical for the oligomerization of LDH and, therefore, also for LDH activity [40]. Although the structure of LDH isoforms is similar, there are differences in their kinetic profiles due to variations in the charged residues surrounding their active sites and variations in the lipophilic residues of the N-terminal tetramerization domains [45].

The ability of LDH to bind NAD(H) is essential to allow substrate entry into the active site. The nucleotide-binding motif known as the Rossmann fold was first identified in LDH, only to be recognized later as a universal structural feature of all NAD(H)-dependent enzymes [40]. A critical hydrogen bond forms between arginine 106 and the substrate carbonyl, which stabilizes the transition state in the hydride-transfer reaction. This seems essential for LDH activity as mutation of Arg106 completely blocks LDH activity [48]. The closure of the flexible active site loop is mainly required for the oxidation of lactate to pyruvate; LDHB should therefore have a better ability to close the mobile loop around Arg106 than LDHA [49]. Catalytic histidine 193 in the substrate-binding domain is another amino acid of crucial importance because it acts as a proton donor to form lactate, and it must be protonated in the LDH-NADH complex before pyruvate binds. Hydride from NADH is then transferred to the carbonyl carbon of pyruvate, and a proton from His193 is transferred to the carbonyl oxygen to complete the addition of H_2_ to give lactate. The pK_a_ that governs the K_m_ for pyruvate is 7.31 for LDHA and 8.26 for LDHB, consistent with variation in the pK_a_ of His193 [47]. Most importantly, the affinity of ligands to the active site of the enzyme is influenced by a different net electrostatic charge arising from a variety of surface residues peripheral to the active sites [47]. The LDHB isoform has a negative net charge (−6) that is consequently characterized by a higher affinity for lactate that preferentially converts it to pyruvate. The LDHA isoform has a positive net charge (+1), which gives it a higher affinity to pyruvate and, as a result, favors the conversion of pyruvate to lactate [47]. However, as reviewed in [50], there are under-appreciated admonitions regarding LDH isozyme functions, which leave the exact in vivo physiological and biochemical roles of LDH isozymes still to be definitively determined, with factors such as metabolic activity, mitochondrial function, or physiologic temperature and binding to other structures or proteins all coming into consideration.

### 3.3. Regulation of LDH Isoforms

#### 3.3.1. LDHA Regulation

Among the three LDH isoforms, LDHA regulation has been studied the most so far (Table 1). The transcription of LDHA is mainly regulated by two transcription factors, i.e., HIF1 and c-MYC (Avian myelocytomatis virus oncogene cellular homolog), which collaborate to activate LDHA transcription in numerous cancer cells [51]. LDHA promoter consists of HRE-A, HRE-B, HRE-C, HRE-D, and HRE-E sites. It has been proven that binding of HIF to the HRE-D site activates LDHA transcription [52]. Increased expression of the proto-oncogene c-MYC, which is involved in the regulation of many cellular processes, e.g., the cell cycle, proliferation, and apoptosis, correlates with increased LDHA expression, stabilization of HIF1 in normoxia, and increased expression of HIF1 in hypoxia. At the same time, LDHA regulates c-MYC by a negative feedback loop mechanism, where inhibition of LDHA increases c-MYC expression [53].

In various types of tumors, the oncogenic transcription factor Forkhead box protein M1 (FOXM1) controls the expression of numerous proteins involved in the regulation of the cell cycle, angiogenesis, and migration of cells. FOXM1 binds to the LDHA promoter, thereby inducing LDHA transcription. In addition, increased expression of FOXM1 leads to increased lactate production [54]. In gastric cancer cells, FOXM1 regulates many cellular processes via LDHA, including stimulation of glycolysis, proliferation, invasion, and migration of cancer cells [55].

The transcription factor Krüppel-like factor 4 (KLF4), which also binds to the promoter region of the LDHA gene, is a negative regulator of LDHA transcription in pancreatic cancer cells. The suppression of KLF4 significantly increased LDHA expression and was correlated with the development and progression of cancer, whereas overexpression of KLF4 inhibited LDHA expression, aerobic glycolysis, and tumor size in in vivo and in vitro models [56].

Epigenetics also play an important role in tumorigenesis and cancer development. It is known that DNA methylation negatively regulates the transcription of LDHA. In immortalized human astrocytes, the silencing of LDHA was associated with increased methylation. In isocitrate dehydrogenase (IDH) mutant gliomas, LDHA was silenced as a consequence of hypermethylation [57].

Various studies have reported that microRNAs (miRNAs) may influence the expression of various genes in cancer cells. In addition, it was shown that miRNAs play a crucial role in cancer metabolism, including glycolysis [58,59]. In colorectal cancer tissues, the expression of LDHA is negatively regulated by miR-34a, miR-34c, miR-369-3p, miR-374a, and miR-4524a/b [60].

Furthermore, experimental evidence suggests that the activity of LDHA is also regulated by post-translational phosphorylation and acetylation of its amino acid residues. The oncogenic receptor tyrosine kinase FGFR1 (fibroblast growth factor receptor 1) phosphorylates LDHA at tyrosine 10 and tyrosine 83. Phosphorylation of Tyr10 and Tyr83 promotes LDHA tetramer formation and binding of NADH to the substrate, which enhances LDHA activity [61].

A recent study has identified another post-translational mechanism of LDHA regulation via protein sirtuin 5 (SIRT5) in prostate cancer. This regulator is a member of the NAD^+^-dependent sirtuin family and could play an important role in the regulation of many cancers. In prostate cancer, SIRT5 is responsible for increased lysine 118 desuccinylation of LDHA. Because succinylated Lys118 positively regulates LDHA enzymatic activity, it is not surprising that low SIRT5 expression was linked to prostate cancer progression. [62].

A different type of regulation involves the acetylation of lysine 5, which promotes the lysosomal degradation of LDHA. SIRT2 is able to deacetylate Lys5 and increase LDHA activity. Interestingly, Lys5 acetylation is decreased in pancreatic cancer [63].

#### 3.3.2. LDHB Regulation

Such as methylation of the LDHA promoter, hypermethylation of the LDHB promoter leads to suppression of LDHB expression (Table 1) and correlates with the metastatic potential of the tumor [64,65]. Methylation of the LDHB promoter is often present in breast cancer cells and plays an important role in cancer development and progression [66]. In tamoxifen resistance of MCF-7 cells, it was observed that changes in LDHB expression were accompanied by demethylation of the LDHB promoter. It seems that LDHB could be involved in breast cancer resistance to chemotherapy and as a potential marker for tamoxifen resistance [67]. Another study reported that the mammalian target of rapamycin (mTOR) was a positive regulator of LDHB expression through the action of the transcription factor STAT3 (signal transducer and activator of transcription 3). The authors suggest that LDHB is a potential treatment target in cancers characterized by aberrant activation of the mTOR signaling cascade [68]. On the other hand, the Krüppel-like transcription factor 14 is a tumor suppressor, which is significantly downregulated in various types of cancers and is involved in numerous processes such as proliferation, differentiation, apoptosis, migration, and invasion of cells. It appears that the downregulation of KLF14 promotes glycolysis via the upregulation of LDHB, whereas overexpression of KLF14 downregulates LDHB expression and glycolysis [69].

In muscle cells, LDHB expression is induced by the peroxisome proliferator-activated receptor coactivator 1α (PGC-1α) through multiple conserved estrogen-related receptor and myocyte enhancer factor 2 binding sites [70].

Interestingly, LDHB also plays a crucial role in lysosomal activity and autophagy, while silencing of LDHB leads to selective inhibition of cancer cell proliferation [71]. During autophagy, LDHB is post-translationally regulated by protein SIRT5, which deacetylates LDHB at lysine 329 and accordingly promotes its activity. In colorectal carcinoma, deacetylated LDHB promotes autophagy and tumor growth [72]. On the other hand, LDHB acetylation or knockout of SIRT5 arrested the autophagic flux [73].

Another post-translational modification of LDHB that affects its activity is serine 162 phosphorylation by Aurora-A, a serine/threonine kinase overexpressed in cancer. Interestingly, this modification increases the activity of LDHB. However, it results in a reaction shift from lactate oxidation to pyruvate reduction and regeneration of NAD^+^ (similar to LDHA), promoting glycolysis, lactategenesis, and tumor growth [74].

#### 3.3.3. LDHC Regulation

The expression of LDHC in cells is suppressed by methylation, similar to how it is for the other two LDH isoforms [75] (Table 1). A recent study demonstrated that demethylation of the LDHC promoter was associated with poor prognosis in patients with breast cancer [76]. However, when it comes to spermatocyte-specific LDHC expression, the CREB (cAMP-response element (CRE)-binding protein) transcription factor seems to play the biggest role [75]. Additionally, an earlier study showed that LDHC expression in mouse non-germ cells was repressed by NF-I (CCAAT box transcription factor) [77].

### 3.4. Role of LDH Isoforms in Carcinogenesis

#### 3.4.1. LDHA in Cancer

LDHA preferentially catalyzes the reversible conversion of pyruvate to lactate and the concurrent regeneration of NADH to NAD^+^, supporting recurring glycolysis. In the human body, LDHA is localized in many tissues, such as the skeletal muscles, blood cells, kidneys, brain, and lungs. During strenuous exercise, LDHA turns pyruvate into lactate and supports glycolysis, which maintains the production of ATP in anaerobic conditions. On the other hand, the abundance of LDHA in serum may serve as a non-specific marker of malignancies. A significantly higher level of LDHA was measured in patients with endometrial adenocarcinoma (349 +/− 100 IU/L) and ovarian cystadenocarcinoma (383 +/− 116 IU/L) in comparison to healthy women (256 +/− 68 IU/L) [78]. The expression of LDHA is increased in cancer cells that prefer glycolytic metabolism, including lymphomas, melanomas, and prostate tumors. Due to LDHA upregulation, tumor cells acquire an aggressive phenotype characterized by cytoskeletal remodeling, angiogenesis, or increased invasiveness and migration. In addition, upregulated LDHA activity and increased production of lactate are associated with resistance to chemotherapy and radiotherapy and lead to poor patient prognosis. Therefore, it is not surprising that LDHA is a promising treatment target. The idea of inhibiting LDH as a potential target in cancer treatment was postulated in the 1960s based on Warburg’s theory [79].

Several small molecule inhibitors of LDH have been developed. However, they did not reach the clinical trial stage because of limited in vivo efficacy, off-target toxicity, or unsuitable pharmacokinetic properties [40]. Additionally, because LDH isoforms are very structurally similar, inhibitors often target both reactions and possibly even other enzymes containing the Rossmann fold. The available inhibitors can be divided into groups by mode of inhibition: substrate-competitive (Oxamate, Compound 24c, PSTMB, 2-amino-5-aryl-pyrazine, Compound 9), nucleotide-competitive (Gossypol, FX11, Quinoline-3-sulfonamide), or inhibitors that compete by occupying both sites (GNE-140, NHI-1, NHI-2, Compound 63) [40]. LDHA-selectivity has been suggested for Quinoline-3-sulfonamides, GNE-140, NHI-1, NHI-2, and Compound 63. Recently, inhibitory peptides that block the tetramerization of LDHA by mimicking the N-terminal domain have been developed [80].

#### 3.4.2. LDHB in Cancer

In contrast to LDHA, the enzyme lactate dehydrogenase B (LDHB) is considered to preferentially catalyze the oxidation of lactate to pyruvate and the concurrent reduction of NAD^+^ to NADH. Compared to LDHA, LDHB is expressed only in certain types of tumors, and its role in tumor progression is not fully understood. Some studies reveal that there is a link between the presence of LDHB and the enhanced proliferation of lung adenocarcinoma and breast cancer [81,82,83]. Increased expression of LDHB in oral squamous cell carcinoma correlates with resistance to taxol leading to poor patient prognosis [84]. High expression of LDHB was also identified in osteosarcoma [85]. Furthermore, the correlation between the overexpression of LDHB and the growth, proliferation, migration, and invasion of osteosarcoma cell lines has been identified. Therefore, increased LDHB expression in patients with osteosarcoma might be a significant prognostic marker for tumor recurrence and poor overall survival [86]. Based on the above-mentioned studies, it appears that LDHB could be an important player in cancer development. So far, AXKO-0046 is the only selective LDHB inhibitor that has been identified. The mechanism behind LDHB inhibition includes the binding of AXKO-0046 to an allosteric site, away from the LDHB catalytic active site, which appears to be critical for its enzymatic activity [87]. Additionally, a macrocyclic peptide (macrocycle 7) that competes with the LDH tetramerization domain has been identified and could serve as a template for the development of more potent LDH disruptors [45].

On the other hand, suppression of LDHB expression promoted the progression of pancreatic cancer and the unfavorable survival of patients with hepatocellular carcinoma [88,89]. Another study examined the correlation between LDHB expression and the serum levels of LDH and showed their prognostic significance in squamous cell carcinoma. Squamous cell carcinoma patients with positive LDHB expression (which correlated with serum LDH) had higher recurrence-free survival in comparison to patients without LDHB expression [90]. Likewise, high LDHB expression is associated with a favorable prognosis in patients with hepatocellular carcinoma, urothelial carcinoma, and prostate cancer [65,88,91].

#### 3.4.3. LDHC in Germ Cells and Cancer

**Figure 5:**
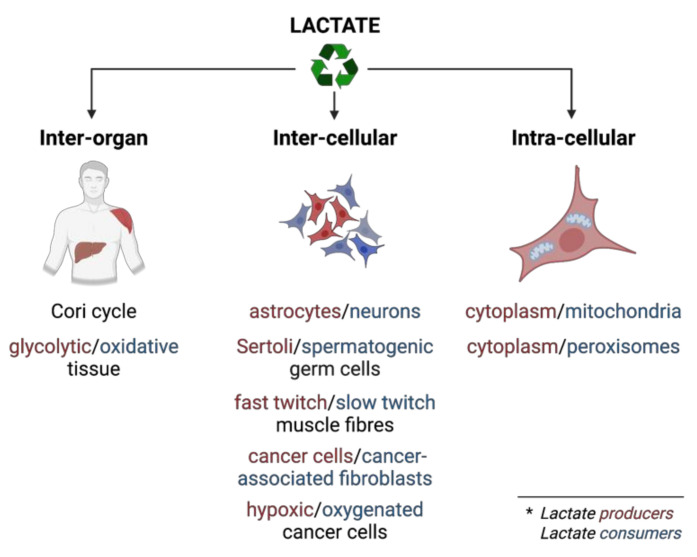
Lactate shuttling. Lactate shuttling happens between tissues, cells, or cellular compartments. (* Lactate-producing cells or compartments are in red, lactate-consuming cells or compartments in blue).

The homotetrameric LDHC isoenzyme is believed to preferentially catalyze the conversion of lactate and NAD^+^ to pyruvate and NADH, such as LDHB. LDHC is a unique isoform of LDH with a characteristic distribution in testicular cells. Originally, it was detected in male spermatozoa and spermatogenic cells and named LDH-X, now known as LDHC [42] (Figure 3). The tissue specificity of LDHC raised the question among scientists about why the testes produce this unique form of LDH and what its role could be [75]. Meiotic and post-meiotic male germ cells depend on the glycolytic metabolism of the somatic Sertoli cells that produce lactate as fuel for germ cells (Figure 5). During spermatogenesis, LDHC thus plays a crucial role in the oxidation of lactate and energy metabolism [92].

Other than the testes, the C isoform of LDH has also been detected in oocytes but at a lower level than in male germ cells [93]. Additionally, LDHC was identified in the blastocyst stage of embryogenesis and was suggested as a maternal genes product that may participate in oocyte maturation or embryonal development [94].

Compared to other LDH isoforms, the role of LDHC in oncogenesis has not been extensively studied. Although it was originally assumed that LDHC was strictly present in the testes, several studies have been published that LDHC is present in a range of cancer tissues, including lung cancer (47%), melanoma (44%), breast cancer (35%), and some prostate cancers [95]. Identification of LDHC as a potential target for anticancer drug discovery has only recently been addressed with (ethylamino)(oxo)acetic acid showing selective inhibition of LDHC in comparison to LDHA or LDHB [96].

## 4. Lactate

### 4.1. Lactate as an Integrating Metabolite and the Role of Monocarboxylate Transporters

The increased buildup of lactate from the LDH reaction hadn’t been previously appreciated for anything other than waste. However, a strategy that produces primarily waste from the majority of consumed fuel would be very inefficient. Moreover, the two most abundant circulating carbon metabolites in the blood stream are glucose (5 mM) and lactate (1 mM), while they can be interconverted in the process of glycolysis and gluconeogenesis [97]. In the tumor microenvironment, lactate concentrations can be as high as 30–40 mM, which is associated with increased metastases and poor patient survival [98]. Apart from being a gluconeogenic precursor, lactate is finally also being recognized as a major oxidative fuel source for the TCA cycle [99]. In fact, tissue TCA cycle labeling from lactate is even greater than that from glucose [100,101]. Lactate has thus been promoted from a waste molecule to the main circulating carbon source for most tissues and tumors and is now perceived as a molecule integrating metabolic pathways to maintain energy/redox homeostasis.

Lactate shuttling [2] or lactate recycling is a form of metabolic cooperation employed at various levels: (a) inter-organ, (b) inter-cellular, and (c) intracellular (Figure 5). Lactate sharing between tissues/cells/cellular compartments involves its production in producer cells or cellular compartments on the one hand and its disposal in consumer cells or cellular compartments on the other. Interestingly, producer cells such as glioblastoma cells convert as much as 90% of taken-up glucose and 60% of taken-up glutamine to lactate [28]. Consumer cells have two options for lactate disposal—the majority is accomplished via oxidation in the TCA cycle, with a minority achieved by gluconeogenesis [102].

Essential components of lactate shuttling are lactate dehydrogenase activity and the monocarboxylate transporters (MCTs) that transport lactate over membranes from producer cells/compartments to consumer cells/compartments. The MCT family has 14 members, of which MCT1-4 are responsible for the proton-linked, bi-directional, passive transport of lactate (as well as of pyruvate and ketone bodies). The correct functioning of MCTs is also facilitated by interacting with chaperone proteins CD147 (basigin) or gp70 (embigin) that anchor MCTs to specific membrane locations [103]. The import and export of lactate depend on the intra- and extracellular concentrations of lactate and protons, although the MCT isoforms also show different affinities for lactate (MCT2 > MCT3 ~ MCT1 > MCT4) or pyruvate (MCT2 > MCT1 > MCT4, 0 for MCT3), turnover rates, and expression pattern over cell types (reviewed in [104]). A classic example of lactate sharing is a hypoxic glycolytic cancer cell that overexpresses MCT4 to export lactate, and an oxygenated oxidative cancer cell that imports the released lactate through MCT1 and uses it for energy production [32]. Despite its low affinity to lactate, MCT4 has a high turnover rate, and its expression is induced in hypoxia, making it well adapted to export lactate along its concentration gradient created by the enormous production of lactate in glycolytic cells, while the ubiquitous MCT1 is able to facilitate lactate uptake into oxidative cancer cells because of their low intracellular lactate levels [33,104]. Similar cooperation occurs in, e.g., glycolytic astrocytes/oxidative neurons, glycolytic Sertoli cells/oxidative spermatogenic germ cells, or fast twitch-glycolytic/slow twitch oxidative muscle fibers. At the organismal level, a typical example is the Cori cycle, where muscle cells produce lactate that is taken up by the liver or kidneys and converted to glucose in the process of gluconeogenesis to support euglycemia. However, as proven by infusing mice with ^13^C-lactate, lactate can be taken up from circulation by virtually any tissue to fuel the TCA cycle [100,101,105,106,107,108]. In contrast to the restricted expression of glucose transporters, the universal expression of MCTs enables the availability of lactate to all cells of the body [97].

**Figure 6:**
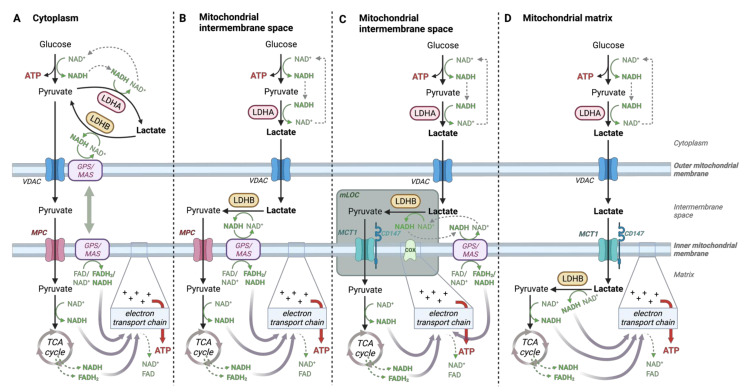
Potential locations of lactate oxidation. (**A**) In case cytosolic LDHB converted lactate into pyruvate in the cytoplasm, lactate carbon would get incorporated into pyruvate and downstream TCA cycle intermediates (pyruvate passes into mitochondria through the voltage-dependent anion channel (VDAC) and the mitochondrial pyruvate carrier (MPC)). However, regenerated NAD^+^ in the LDHA reaction would get consumed again by LDHB unless glycerol-3-phosphate (GPS) or malate-aspartate (MAS) shuttles supplied NAD^+^ and moved reducing equivalents into mitochondria to be used in the electron transport chain. (**B**) Such as pyruvate, lactate can cross the outer mitochondrial membrane through VDAC. LDHB in the mitochondrial intermembrane space would thus save LDHA-regenerated NAD^+^ in the cytosol but reducing power from lactate would have to be translocated into the mitochondrial matrix again with the help of GPS/MAS because of mitochondrial membrane impermeability to NAD(H) [113]. (**C**) This option shows the mitochondrial lactate oxidation complex (mLOC) [111] at the outer side of the inner membrane, consisting of LDHB, monocarboxylate transporter 1 (MCT1), its chaperone CD147, and cytochrome oxidase (COX) leading to similar fluxes as in (**B**). (**D**) If MCT1 transported lactate into the mitochondrial matrix, matrix LDHB could oxidize it to pyruvate and supply reducing equivalents for the electron transport chain directly, independent of the GPS or MAS [4,109].

Lactate recycling in the TCA cycle previously implied its conversion to pyruvate in the cytoplasm by LDHB with the subsequent import of the lactate-derived pyruvate into mitochondria for further oxidation (Figure 6A). However, another concept had also emerged considering the mitochondrion as a pyruvate sink, promoting lactate oxidation to pyruvate in this compartment. In this model, lactate is imported into mitochondria directly, where it is converted to pyruvate by mitochondrial LDHB. The model was confirmed by the oxidation of ^13^C-lactate in isolated mitochondria and by the detection of LDHB and MCT1 in mitochondria [4,109,110,111]. Mitochondrial lactate oxidation to pyruvate is now widely accepted, and the only controversy remaining is as to the exact location of this reaction within the mitochondrion [112]. Some groups claim that mitochondrial conversion of lactate to pyruvate happens in the inter-membrane space [50,111,113] (Figure 6B, C), while others believe this reaction takes place directly in the matrix [4,109] (Figure 6D). In any case, we can conclude that lactate is the end-product of both anaerobic and aerobic glycolysis and is capable of linking glycolysis to oxidative phosphorylation in the presence of oxygen [50]. TCA cycle entry through the irreversible PDH reaction generates acetyl-CoA, which cannot be reconverted back to pyruvate. It, therefore, means that PDH also irreversibly clears lactate and indirectly controls lactate oxidation [97]. Unlike LDH, PDH is highly regulated allosterically and by covalent modifications (phosphorylation by PDHKs), giving more room to control. Interestingly, high PDH activity characterizes lactate-consuming neurons in culture [113].

### 4.2. Lactate-Pyruvate Shuttle and Redox Homeostasis

Mitochondrial lactate oxidation makes sense not only because lactate could provide extra carbon on top of pyruvate for the TCA cycle. Lactate is basically a reduced form of pyruvate, which means it also carries extra electrons that could influence mitochondrial bioenergetics through the supply of NADH, which is essential for the electron transport chain and, ultimately, for ATP generation. However, the outer and inner mitochondrial membranes are impermeable to NAD^+^ and NADH, so whether lactate is oxidized in the mitochondrial intermembrane space or the matrix makes a difference. The indirect transfer of NADH through the mitochondrial membranes depends on the well-known malate-aspartate (MAS) and glycerol-3-phosphate (GPS) shuttles, but it seems that the reversible LDH reaction could also serve as another important electron shuttle.

NADH is formed during glycolysis in the cytosol by glyceraldehyde-3-phosphate dehydrogenase. For continual glycolysis, NAD^+^ needs to be regenerated, usually by the LDHA reaction, completing a redox-neutral cycle. Oxidation of lactate back to pyruvate in the cytosol by cytosolic LDHB wouldn’t thus support the objective of recycling NADH to NAD^+^ for ongoing glycolysis because NAD^+^ would be consumed again (Figure 6A). A benefit to having mitochondrial LDHB is that it allows a glycolytic cell to use its own lactate as a source of carbon and reducing equivalents directly inside mitochondria without compromising the cytosolic NAD^+^ regeneration. Moreover, such an arrangement would efficiently maximize the energy yield from the breakdown of glucose by enabling NADH generated by LDHB to be passed to Complex I of the electron transport chain [100].

Lactate most likely crosses the outer mitochondrial membrane through the voltage-dependent anion channel (VDAC), such as pyruvate [114,115]. In case of the presence of mitochondrial LDH in the intermembrane space, NADH generated during the conversion of lactate to pyruvate would have to be translocated into the mitochondrial matrix with the help of the malate-aspartate shuttle (Figure 6B). Such a “lactate-malate-aspartate shuttle” would couple lactate oxidation to the malate-aspartate shuttle in the intermembrane space to translocate reducing power to the matrix [113]. In this arrangement, the LDHB position near a mitochondrial pyruvate carrier would be beneficial in decreasing the local concentration of pyruvate which could help promote lactate oxidation. A mitochondrial lactate oxidation complex (mLOC) has been discovered in this compartment composed of LDHB in the intermembrane space tethered to MCT1 interacting with its chaperone CD147 (basigin) and cytochrome oxidase (COX; Complex IV of the electron transport chain) in the inner membrane (Figure 6C) [111].

If lactate oxidation occurred directly in the mitochondrial matrix (Figure 6D), the putative matrix LDH and MCT would enable lactate to carry its reducing power into the mitochondria independent of the malate-aspartate shuttle [4,109]. Mitochondrial LDH and MCT are confirmed in the MitoCarta database, but their precise location within mitochondria is unknown. Nevertheless, MCT1 colocalizes with Complex IV (cytochrome oxidase) of the electron transport chain embedded within the inner mitochondrial membrane, which suggests the possibility of lactate transport through the inner membrane into the matrix [2], even though the idea is criticized for being incompatible with the cytoplasmic/matrix redox gradient, which could dissipate the proton motive force. [112]. Possibly, short-term matrix lactate oxidation could be favored by the rapid clearing of pyruvate by the PDH complex. Consequently, the purpose of matrix lactate oxidation may be to offer a mechanism for carrying reducing equivalents produced by glycolysis into the mitochondria, compensating for limitations in the glycerol phosphate and malate-aspartate shuttles when the glycolytic flux is increased [4]. Although, the cytosolic-to-mitochondrial shuttles may work in parallel to balance the redox state. Interestingly, in cases of inactivating LDH mutations or upon inhibition of LDHA, excess glycerol-3-phosphate is generated, suggesting that the glycerol-3-phosphate shuttle activity compensates for LDH loss of function. Conversely, simultaneous loss of LDH and glycerol-3-phosphate dehydrogenase disrupts ATP homeostasis and glycolytic flux in the developing *Drosophila larvae* [116].

### 4.3. Tumor Microenvironment Acidity Coupled to Lactate Production and Transport

Glucose metabolism is a continuous source of acids. In order to keep up with the metabolic rate, tumors must establish an adequate means of clearing their acidic end-products [117], while an acidic tumor microenvironment causes the breakdown of the extracellular matrix, promotion of new vessel formation, and suppression of the immune response, which are characteristics associated with aggressive tumors and increased metastases [118]. Glycolysis and the TCA cycle generate a large flow of lactate with a stoichiometric amount of protons, and CO_2_, respectively [119]. Protons are produced from the consumption of glycolysis-derived ATP by ATPases and kinases, and together with lactate, they can form lactic acid at lower extracellular pH_e_ of the tumor microenvironment [119]. The pKa of lactic acid is 3.8, meaning that at physiologic pH, lactate stays in its deprotonated form as the lactate anion and therefore requires a transporter for its movement across membranes, such as the MCTs that facilitate H^+^-linked lactate transport (discussed in more detail in Section 4.1). Interestingly, MCT1 and MCT4 have also been reported to form a metabolon with the transmembrane carbonic anhydrase IX (CAIX; another HIF-1 target gene [120] and a pH regulator catalyzing the reversible hydration of CO_2_ to bicarbonate and proton [121]) in breast cancer cells (and not healthy tissue) through its binding to the CD147 chaperone [122]. Analogously, the membrane-anchored extracellular carbonic anhydrase IV (CAIV) has also been found to interact with MCTs through the CD147 or gp70 chaperones [123]. In the case of CAIX, the connection to lactate metabolism seems to be multilayer because lactate has been found to stimulate its expression in normoxia [124], while conversely, CAIX is required for the maximal expression and activity of LDHA [125].

Compared to normal tissues, where pH_i_ (intracellular pH) and pH_e_ (extracellular pH) are about 7.2–7.4, tumors are able to maintain their pH_i_ at around 7.4, while their pH_e_ falls to around 6.5 [126]. Lactate itself does not impact the pH of cells or their microenvironment. However, the protons cotransported with lactate influence pH_i_ intracellularly and pH_e_ extracellularly, while at low pH_e_, as already mentioned, lactic acid may form. A suitable equilibrium between intracellular pH and intracellular lactate retention must be therefore achieved by the system. Interestingly, the lactate and proton (lactic acidosis) present in the tumor microenvironment together protect cancer cells from glucose deprivation. However, lactosis (high lactate concentration, pH 7.4) and acidosis (low lactate concentration, pH 6.6) separately do not protect cells against limited glucose supply [127]. Non-specific mechanisms of lactic acidosis have been suggested to foster this effect but maybe the fact that lactate can serve as an oxidative fuel, while at the same time, low pH inhibits glycolysis, plays the biggest role in surviving glucose deprivation.

### 4.4. Lactate-Induced Posttranslational Protein Modifications

There is growing evidence that metabolic intermediates and end products may have signaling functions in addition to their metabolic roles. A well-known example is acetyl-CoA, which can be used by histone acetyltransferases (such as p300 or CBP) for lysine acetylation, increasing gene expression by relaxing the chromatin and exposing sites in the DNA for transcription factors [128]. Interestingly, lactate provides carbons for acetyl-CoA and subsequent histone acetyl-residues in glioblastoma this way, affecting gene expression through modulation of the epigenome in a manner dependent on oxidative metabolism and the ATP-citrate lyase [129].

In 2019, lysine lactylation was described as a new post-translational protein modification [130]. The authors showed that lactate-derived lactylation of histone lysine residues served as an epigenetic modification that directly stimulated gene transcription. Exogenous lactate, increased glycolysis, or hypoxia all induced lysine-lactylation. Deletion of both LDHA and LDHB fully suppressed the production of lactate and histone lysine-lactylation [130]. Interestingly, lactate was transformed into lactyl-CoA and transferred onto histones by acetyltransferases (such as p300) and removed by histone deacetylases HDAC 1-3 and SIRT 1-3 [131]. In non-small cell lung cancer, lysine-lactylation sites were identified directly in the promoters of hexokinase-1 and isocitrate dehydrogenase, downregulating and upregulating their expression, respectively [132]. Additionally, in gastric cancer cells, more than 2000 sites for lysine-lactylation in non-histone proteins were identified, while lactylated lysines influenced RNA splicing and were more abundant in gastric tumors in comparison to adjacent tissue [133].

Even before the discovery of epigenetic lysine lactylation, lactate-facilitated escape from proteasomal degradation had been described [134]. In this elaborate study, the authors reported a hypoxic response that depended on the accumulation of lactate and was independent of HIF. They found that the NDRG3 protein (implicated in cell migration and invasion) was degraded in a PHD2/VHL-dependent manner in normoxia but was protected from ubiquitination and subsequent destruction by being labeled with lactate that accumulated under prolonged hypoxia.

### 4.5. Lactate Receptor

In recent years, it has also become clear that metabolites may function similarly to hormones or neurotransmitters as extracellular signaling molecules through G-protein coupled receptors [135]. Specifically, lactate can signal through hydroxycarboxylic acid receptor 1 (HCAR1), also known as G-protein coupled receptor 81 (GPR81) [136]. Lactate activation of GPR81 results in cAMP downregulation, leading to a decrease in intracellular cAMP concentrations. GPR81 is mainly expressed in adipose tissue, where lactate signaling results in the inhibition of lipolysis [137]. However, GPR81 has also been found to be highly expressed in various tumors and cancer cell lines. In pancreatic cancer, GPR81 correlated with tumor growth and metastasis [138]. Interestingly, lactate addition to culture media increased the expression of lactate metabolism genes, including the MCTs, but not in GPR81-silenced cells [138]. GPR81 is also induced in breast cancer cells, where its silencing inhibits cell proliferation, migration, and model tumor growth [139]. Glycolysis and ATP production were also decreased in cells with knocked-down GPR81. Additionally, clinical samples of breast cancer demonstrated high GPR81 expression compared to that of normal breast tissues [139]. It, therefore, seems that GPR81 is an important regulator of lactate signaling in cancer and another possible target for anti-cancer therapy.

## 5. Conclusions

After decades of confusion over why cancer cells waste so much carbon from consumed glucose, lactate has finally received the recognition it deserved all along. From the pioneers of exercise physiology [102], lactate significance is currently recognized also in the field of cancer metabolism. It has even been postulated that lactagenesis in cancer could be the purpose and explanation of the Warburg effect [140]. In the context of the Warburg effect, it is now understood that lactate acts as a metabolic substrate, as well as a signaling molecule, promoting multiple hallmarks of cancer as a powerful regulator [141]. By linking the metabolic cellular state to gene expression through posttranslational histone modifications, lactate also facilitates cell adaptation to the new environment [142]. In order to take advantage of lactate’s crucial involvement in cancer metabolism, numerous treatment strategies targeting lactate metabolism and transport have been created. Even though small molecule inhibitors have demonstrated encouraging efficacy, these therapeutic approaches still face many challenges, and further research is required to make them clinically relevant [143]. On the other hand, AZD3965, a second-generation MCT1 inhibitor, has already reached the Phase I/II clinical trial stage for patients with various types of tumors [144]. Repurposing older molecules could also prove beneficial, such as in the case of 5-ALA (δ-aminolevulinic acid), an FDA-approved drug that accumulates heavily in glioblastomas and has been found to inhibit LDH [145].

## Figures and Tables

**Table 1:** Regulation of LDH isoforms at the level of transcription, protein, and activity (more details in text).

Regulation at the Level of:		LDHA		LDHB		LDHC	
		Induction	Repression	Induction	Repression	Induction	Repression
RNA	Epigenetically		methylation		methylation		methylation
	Transcription factors	HIF MYC FOXM1	KLF4	STAT3 PGC-1α	KLF14	CREB	NF-I
Protein	Translation		miRNA				
	Degradation	SIRT2 (K5 deacetylation)	K5 acetylation				
Activity	Post-translational modifications	FGFR1 (Y10, Y83 phosphorylation) K118 succinylation	SIRT5 (K118 desuccinylation)	SIRT5 (K329 deacetylation) Aurora-A (S162 phosphorylation)	K329 acetylation		
